# Gut microbiota-derived trimethylamine N-oxide and the incidence and recurrence of atrial fibrillation: a systematic review and meta-analysis

**DOI:** 10.3389/fcvm.2026.1897722

**Published:** 2026-08-24

**Authors:** Jie Dong, Lei Chen, Nan Zheng, Ming Yang

**Affiliations:** Department of Cardiovascular Surgery, Institute of Cardiac Surgery, The Sixth Medical Center of PLA General Hospital, Beijing, China

**Keywords:** atrial fibrillation, biomarkers, cardiovascular diseases, gastrointestinal microbiome, meta-analysis, trimethylamine N-oxide

## Abstract

**Objective:**

To quantify the association between circulating trimethylamine N-oxide (TMAO) and atrial fibrillation (AF) risk and determine how this association is modified by clinical background.

**Methods:**

We performed a systematic review and meta-analysis by searching PubMed, Web of Science, EMBASE, Scopus, and ProQuest for studies published up to April 2026. Studies reporting quantitative associations between circulating TMAO levels and AF outcomes were included. Meta-analytic pooling of odds ratios (ORs) and hazard ratios (HRs) was conducted using R packages meta under random-effects models. Pre-specified subgroup analyses were performed by clinical setting and AF outcome type. Heterogeneity was quantified using I^2^ statistics and *τ*^2^, with influence diagnostics and assessment of publication bias using Galbraith plots, Baujat plots, and Egger's test.

**Result:**

Of the 15 included studies, 9 were included in the quantitative meta-analysis, comprising 8 odds-ratio and 2 hazard-ratio studies with one overlapping. The random-effects meta-analysis of the eight odds ratio studies demonstrated a significant positive association between TMAO and AF (pooled OR 1. 35, 95% CI 1. 15–1. 59, *p* = 0. 0003). Substantial heterogeneity was observed (I^2^ = 68.3%, *p* = 0Subgroup analysis revealed that only 1 of the 8 studies (12. 5%) reported postoperative AF after cardiac surgery, in which the association was significantly stronger (OR 2. 88, 95% CI 1. 35–6. 15) compared with spontaneous incident AF (pooled OR 1. 31, 95% CI 1. 12–1. 52; between-group *p* = 0. 045). A trim-and-fill sensitivity analysis adjusting for potential publication bias attenuated the pooled estimate (OR 1.18, 95% CI 0.95–1.47). Analysis of two studies reporting hazard ratios showed extreme heterogeneity (I^2^ = 92.5%) precluding a reliable pooled estimate.

**Conclusion:**

Circulating TMAO is significantly associated with increased AF risk, with the magnitude of risk markedly augmented in the postoperative cardiac surgery setting,positioning TMAO as a context-dependent biomarker that requires interventional evidence before being considered a target for gut microbiota-directed interventions.

## Highlights

- TMAO is associated with 35% increased odds of atrial fibrillation (OR 1.35).
- The TMAO-AF association exhibits substantial heterogeneity (I^2^ = 68.3%).
- Postoperative AF risk is nearly tripled with elevated TMAO (OR 2.88).
- TMAO may serve as a key target for probiotics modulating the gut-heart axisTMAO represents a candidate biomarker for evaluating probiotics’ effects on the gut-heart axis, pending interventional studies.

## Introduction

AF is the most prevalent sustained cardiac arrhythmia worldwide, imposing a substantial burden through increased risks of stroke, heart failure, and all-cause mortality. The pathogenesis of AF is multifactorial, with emerging evidence underscoring the critical role of the gut-heart axis in cardiovascular disease progression. Within this paradigm, the gut microbiota-derived metabolite TMAO has garnered significant attention as a novel risk marker (1) and potential therapeutic target. TMAO is primarily produced through the gut microbial metabolism of dietary choline and L-carnitine, which are abundant in red meat, eggs, and dairy products. Dietary modification, particularly the reduction of meat intake, has been shown to lower circulating TMAO levels and may represent a practical strategy for mitigating cardiovascular risk. Probiotics, which modulate gut microbial composition, may similarly influence cardiovascular outcomes by targeting TMAO production, positioning TMAO as a candidate biomarker that may inform microbiota-directed interventions in AF prevention and management (2).

The association between circulating TMAO levels and AF risk has been quantitatively assessed in prior syntheses. A systematic review and meta-analysis (3) reported a pooled odds ratio of 1.40 for AF incidence with higher TMAO concentrations, derived from six studies with low between-study heterogeneity (I^2^ = 19.8%). This finding suggested a consistent, population-agnostic effect, reinforcing TMAO's potential as a universal biomarker for AF risk stratification. However, the included studies encompassed relatively homogeneous cohorts, and the analysis did not extensively evaluate effect modifiers across diverse clinical settings, leaving open questions about the generalizability of this association.

Notably, TMAO's role in AF pathophysiology may be context-dependent, influenced by underlying comorbidities, renal function, and procedural interventions such as cardiac surgery. The gut microbiome, which governs TMAO production, exhibits considerable interindividual variability, further suggesting that the TMAO-AF relationship might be modulated by specific clinical backgrounds (2). Consequently, the low heterogeneity observed in earlier meta-analyses may mask important subgroup differences, necessitating a stratified approach to elucidate which patient populations are most susceptible to TMAO-associated AF risk. Such insights are essential for advancing targeted strategies, including probiotic supplementation aimed at mitigating TMAO-related pathways.

To address these gaps, we conducted an updated systematic review and meta-analysis of prospective studies examining the association between TMAO and AF. This study specifically investigates the substantial heterogeneity across clinical contexts through pre-specified subgroup analyses based on disease background and AF outcome type. Our aims are to provide a refined quantitative synthesis of the TMAO-AF association, to identify and quantify sources of between-study variability, and to delineate clinical settings where this relationship is most pronounced, thereby informing future mechanistic research and precision-based interventions.

## Methods

### Data sources and search strategy

A systematic literature search was conducted across five electronic databases: PubMed, Web of Science, EMBASE, Scopus, and ProQuest. The search strategy aimed to identify studies investigating the association between TMAO and AF. No date restrictions were applied, and the final search was updated on April 2026. The search combined Medical Subject Headings (MeSH) terms and keywords related to “TMAO,” “trimethylamine N-oxide,” “gut microbiota,” and “atrial fibrillation.”

The PubMed search strategy was:

(“Trimethylamine N-oxide"[Mesh] OR “Trimethylamine N-oxide"[tiab] OR TMAO[tiab] OR “gut microbiota metabolite"[tiab]) AND (“Atrial Fibrillation"[Mesh] OR “Atrial Fibrillation"[tiab] OR AF[tiab] OR “auricular fibrillation"[tiab])

Searches in other databases used analogous terms with platform-specific syntax. Reference lists of included studies and relevant reviews were manually screened to identify additional publications. The study selection followed the Preferred Reporting Items for Systematic Reviews and Meta-Analyses (PRISMA) guidelines (4).

### Eligibility criteria

Studies were selected based on pre-defined PICO criteria. Population: Adult human populations (aged ≥18 years). Exposure: Measurement of circulating TMAO levels. Comparator: Lower levels or categories of TMAO.

Outcomes: The primary outcome was atrial fibrillation occurrence (incident, postoperative, or post-ablation recurrence). Secondary outcomes included AF subtypes and relevant composite endpoints.

Study Design: Observational studies (cohort, case-control, cross-sectional) and randomized controlled trials reporting relevant outcomes were included. Reviews, meta-analyses, editorials, case reports, and animal studies were excluded.

Other Criteria: Only English-language studies reporting quantifiable effect estimates (e.g., odds ratio, hazard ratio) with measures of precision were included.

### Data extraction and quality assessment

Two reviewers independently screened titles, abstracts, and full texts. Discrepancies were resolved by consensus or by consulting a third reviewer. Data were extracted using a standardized form, capturing study characteristics, population details, TMAO measurement method, AF definition, follow-up, effect estimates, and adjusted covariates.

Two reviewers independently assessed the methodological qualityFor risk of bias assessment, the Cochrane Risk of Bias 2. 0 tool was used for the single randomized controlled trial (5), and the Risk Of Bias In Non-randomized Studies of Interventions (ROBINS-I) tool was applied to the seven observational studies (6). We acknowledge that ROBINS-I is primarily designed for intervention studies, but we considered it acceptable because our included studies involved quantifiable exposure levels; however, we note this as a limitation. The robvis tool was used to visualize risk-of-bias assessments (7).

### Statistical analysis

Analyses were performed using R software (version 4.3.0) with the meta (8) and metafor packages. For studies with multiple estimates, the most fully adjusted model was used. Continuous estimates and studies with different TMAO thresholds were not combined in the same analysis. Categorical estimates (highest vs. lowest TMAO quantile) were used for the primary meta-analysis. Study-specific log odds ratios or log hazard ratios and their standard errors were pooled using inverse-variance weighting. Heterogeneity was quantified using the I^2^ statistic and Cochran's *Q* test (*p* < 0.10). A random-effects model (restricted maximum-likelihood estimator) was used as the primary model, with a common-effect model for comparison. Results were presented in forest plots. We acknowledge the clinical and biological diversity of AF outcomes. To provide a comprehensive assessment while accounting for this heterogeneity, we defined the primary outcome as AF occurrence of any clinical subtype (incident AF, postoperative AF, AF recurrence, AF presence/progression, and prognostic outcomes in established AF) and subsequently performed subgroup analyses by AF outcome type to quantify effect modification. Pre-specified subgroup analyses explored heterogeneity by clinical setting and AF outcome type, tested via meta-regression. Sensitivity analysis used the leave-one-out method and was visualized with a Baujat plot. Publication bias was assessed via funnel plots, Begg's test (9), and Egger's test (10), with further investigation using the trim-and-fill method. A Galbraith plot assessed individual study contributions to heterogeneity. A supplementary meta-analysis of hazard ratios was conducted where applicable. A two-sided *p*-value < 0.05 denoted statistical significance, except for heterogeneity tests (*p* < 0.10).

## Results

### Study selection process

**Figure 1:**
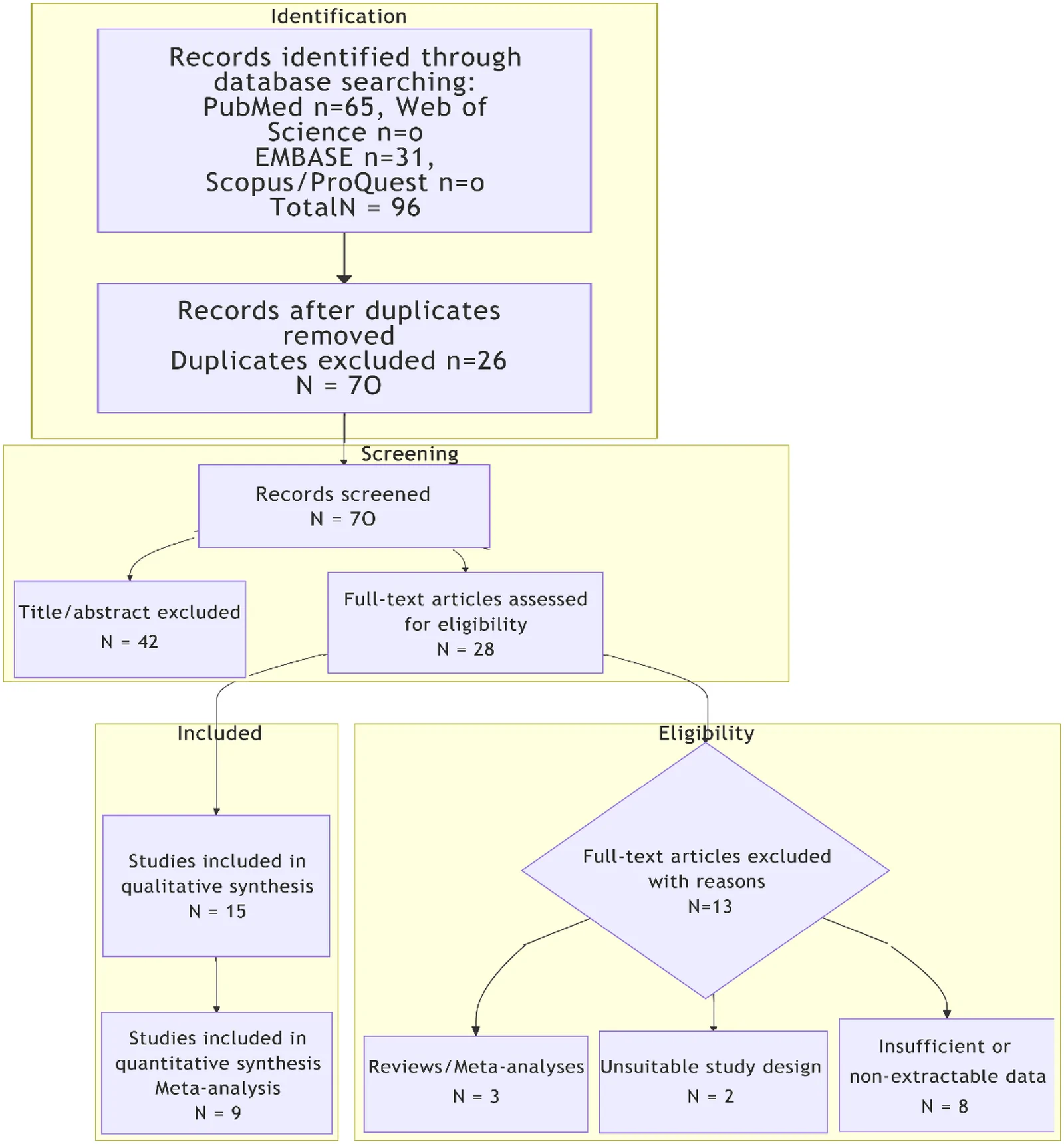
PRISMA flow diagram of study identification, screening, eligibility assessment, and inclusion. The diagram shows that 15 studies were included in qualitative synthesis, 9 in quantitative synthesis (8 contributed to odds ratio meta-analysis, 2 to hazard ratio meta-analysis; one study contributed to both analyses). Flow diagram depicting the systematic literature search and study selection process according to the Preferred Reporting Items for Systematic Reviews and Meta-Analyses (PRISMA) guidelines.

A total of 96 records were initially identified through systematic searches of PubMed, Web of Science (*n* = 65), EMBASE, Scopus, and ProQuest (*n* = 31). After removal of 26 duplicate records, 70 unique citations were screened based on title and abstract, of which 42 were deemed irrelevant and excluded. The remaining 28 full-text articles were assessed for eligibility against pre-specified inclusion and exclusion criteria. Of these, 13 studies were subsequently excluded for the following reasons: reviews or meta-analyses (*n* = 3), unsuitable study design (*n* = 2), and insufficient or non-extractable data (*n* = 8). Ultimately, 15 studies were retained for qualitative synthesis. Of these 15, 9 studies provided adequate effect estimates suitable for quantitative meta-analysis. Specifically, 8 studies contributed odds ratios to the primary OR meta-analysis (11–18), while 2 studies contributed hazard ratios to a supplementary HR meta-analysis (13, 19). Because Svingen et al. (13) provided both OR and HR estimates, it was included in both analyses, yielding 9 independent studies across the two quantitative syntheses. The remaining 6 studies from the qualitative synthesis were excluded from quantitative pooling because they employed cross-sectional or case-control designs without longitudinal AF risk estimates, reported only group-level comparisons without estimable effect sizes, or assessed endpoints other than incident or recurrent AF. The complete study selection workflow is illustrated in Figure 1.

### Characteristics of included studies

The 15 studies eligible for qualitative synthesis, summarized in Table 1, encompassed a broad spectrum of clinical settings and geographic regions, with sample sizes ranging from 65 to 14,846 participants. Study designs were predominantly prospective cohorts (k = 10), supplemented by retrospective cohorts (k = 2), cross-sectional analyses (k = 2), and one nested case-control study. The underlying disease contexts varied considerably, including established AF, heart failure with reduced or preserved ejection fraction, post-myocardial infarction chronic heart failure, end-stage kidney disease, suspected stable angina, and postoperative cardiac surgery. Follow-up duration ranged from in-hospital assessment to a median of 10.9 years across studies reporting longitudinal outcomes. A detailed extraction table (Supplementary Table S1) is provided, documenting for each of the 15 studies the exact outcome definition, TMAO exposure contrast, effect estimate, adjustment covariates, and whether the study was included in the OR or HR quantitative analysis. Among the 9 studies in the quantitative synthesis, 8 contributed to the primary OR meta-analysis and 2 to the supplementary HR meta-analysis, with one study (13) overlapping between the two analyses. Primary endpoints were heterogeneous, encompassing incident AF, AF recurrence after catheter ablation, all-cause and cardiovascular mortality, and composite major adverse cardiovascular events.

**Table 1:** Characteristics of included studies.

Study	Country	Study Design	Sample Size	Disease/Clinical Setting	Primary Endpoint	Follow-up
Suzuki et al., 2016 (11)	UK	Retrospective cohort	972	Acute heart failure	All-cause mortality and death or HF rehospitalization at 1 year	1 year
Zuo et al., 2018 (12)	Norway	Prospective cohort	14,846	Atrial fibrillation	AF hospitalization or death	Median 10.9 years
Svingen et al., 2018 (13)	Norway	Prospective cohort	3,797	Suspected stable angina	Incident atrial fibrillation	Median 7.3 years
Liang et al., 2019 (20)	China	Retrospective cohort	179	AF with or without ischemic stroke	Association of TMAO with ischemic stroke in AF patients	N/A
Stubbs et al., 2019 (14)	USA	Post hoc analysis of RCT	1,243	ESKD on hemodialysis	Cardiovascular composite (death, MI, stroke, PVE, HUA)	64 months
Gong et al., 2019 (21)	Romania	Cross-sectional	154	Cardiovascular disease	Association of serum TMAO with AF occurrence	N/A
Zhou et al., 2020 (15)	China	Prospective cohort	1,208	CHF after MI	MACE (all-cause mortality, HF rehospitalization, recurrent MI)	Median 672 days
Büttner et al., 2020 (22)	Germany	Prospective cohort	65	Atrial fibrillation	TMAO levels across AF progression phenotypes	12–18 months
Papandreou et al., 2021 (16)	Spain	Nested case-control (PREDIMED trial)	1,879	High cardiovascular risk	Incident atrial fibrillation	∼10 years
Kinugasa et al., 2021 (17)	Japan	Prospective cohort	146	HFpEF	Composite of cardiac death and HF hospitalization	Mean 872 days
Nguyen et al., 2021 (23)	Netherlands	Prospective cohort	78	Atrial fibrillation	TMAO levels by AF subtype	N/A
Luciani et al., 2023 (24)	Switzerland	Prospective cohort	2,379	Atrial fibrillation	Cardiovascular mortality	Median 4.0 years
Nenna et al., 2024 (18)	Italy	Prospective cohort	100	Postoperative AF (cardiac surgery)	Incidence of postoperative AF	Until hospital discharge
Meng et al., 2024 (19)	China	Prospective observational	152	Atrial fibrillation	AF recurrence after catheter ablation	1 year
Florea et al., 2025 (25)	Romania	Cross-sectional	154	CVD with/without AF	Association of serum TMAO with AF presence	N/A

AF, atrial fibrillation; CHF, chronic heart failure; CKD, chronic kidney disease; CVD, cardiovascular disease; ESKD, end-stage kidney disease; HF, heart failure; HFpEF, heart failure with preserved ejection fraction; HUA, hospitalization for unstable angina; MACE, major adverse cardiovascular events; MI, myocardial infarction; N/A, not applicable; PVE, peripheral vascular event; RCT, randomized controlled trial; TMAO, trimethylamine N-oxide.

### Risk of bias assessment

**Figure 2:**
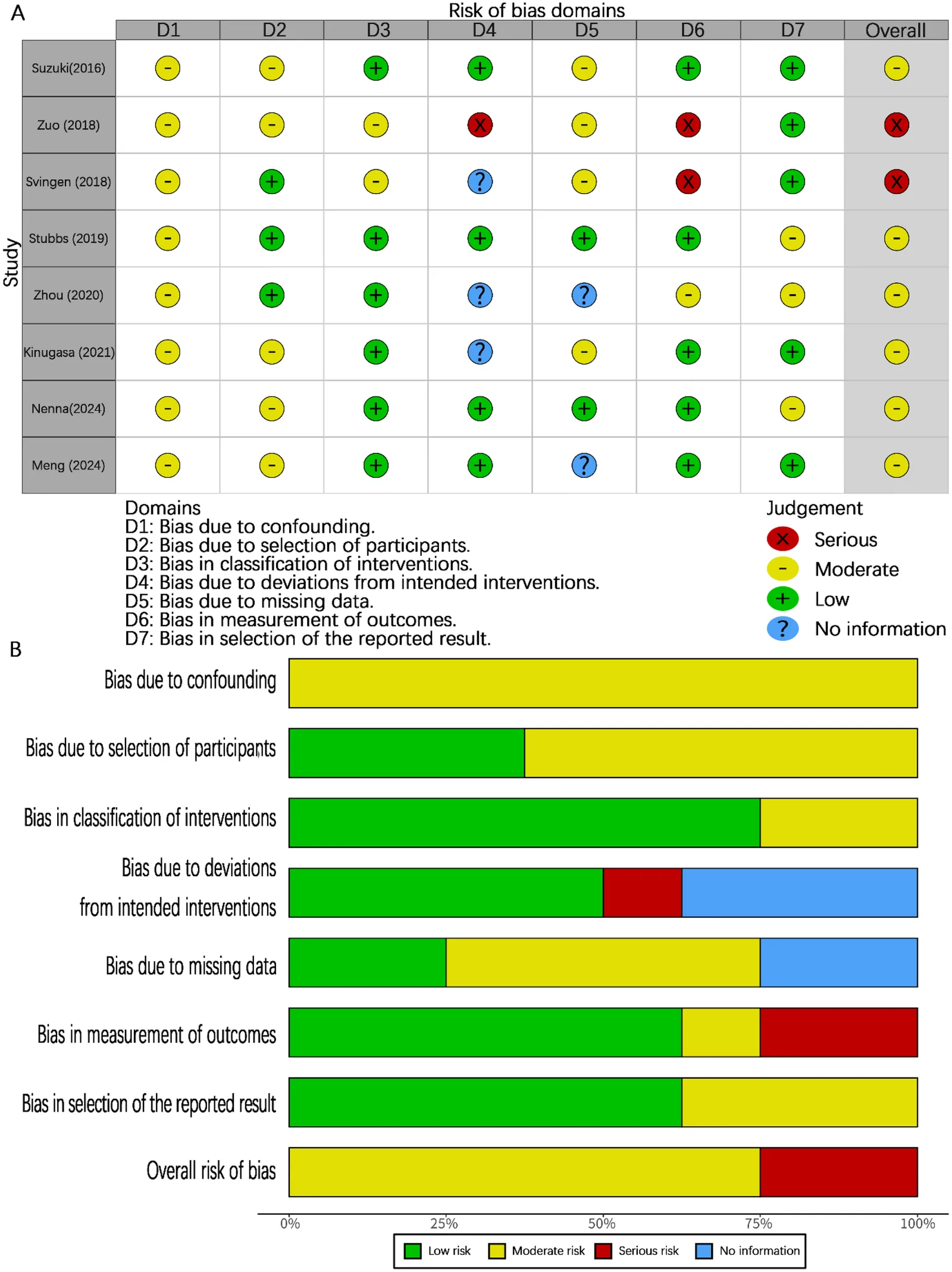
Summary of risk of bias assessment for included studies. Risk of bias summary for the eight studies included in the quantitative meta-analysis. **(A)** ROBINS-I traffic light plot of domain-specific risk of bias for individual non-randomized studies. **(B)** ROBINS-I summary bar plot of cumulative risk-of-bias distribution across domains.

The methodological quality and risk of bias of the included studies were formally appraised using domain-specific tools appropriate for each study design. The single randomized controlled trial (16) was evaluated with the Cochrane Risk of Bias 2.0 tool and was judged to be at low risk of bias across all six assessed domains (randomization process, deviations from intended interventions, missing outcome data, outcome measurement, selection of reported result, and overall bias). The remaining seven non-randomized observational studies were assessed using the Risk of Bias In Non-randomized Studies of Interventions (ROBINS-I) tool. As summarized in Figure 2, the majority of these studies were rated as having moderate overall risk of bias, with one study (12) receiving a serious overall rating due to concerns arising from confounding and selection of participants. The most frequently downgraded domains were confounding and selection of participants, primarily attributable to the inherent limitations of observational designs and incomplete adjustment for established AF risk factors. We recognize that the ROBINS-I tool is primarily intended for non-randomized intervention studies, but we applied it to the observational studies in this meta-analysis because they examined the association between TMAO exposure and AF outcome, which is analogous to an intervention effect in terms of between-group comparison. For the Papandreou et al. nested case-control analysis within the PREDIMED randomized trial, we used RoB 2. 0 because the parent study was a randomized controlled trial, and we assessed the risk of bias for the specific nested analysis within that context. We acknowledge that this application may be debated, and we have noted this limitation in the discussion section. Importantly, no study was rated as being at critical risk of bias, and the domains of measurement of outcomes and missing data were generally well addressed.

### Overall meta-analysis of TMAO and AF risk

The meta-analysis incorporated eight prospective cohort studies comprising a total of k = 8 independent risk estimates for the association between circulating TMAO concentrations and incident or postoperative AF. Under a common-effect framework, the pooled OR was 1.17 (95% CI, 1.11–1.22; z = 6.63, *p* < 0.0001), whereas the random-effects model, which accounts for between-study variance, yielded an OR of 1.35 (95% CI, 1.15–1.59; z = 3.65, *p* = 0.0003).

**Figure 3:**
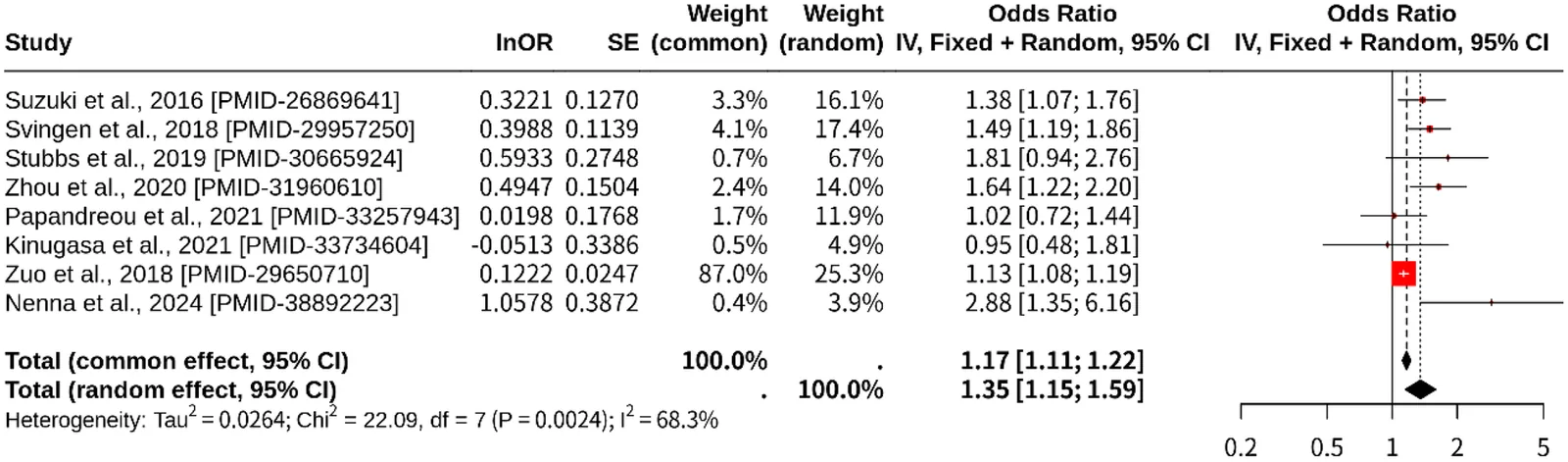
Forest plot of the odds ratio meta-analysis of TMAO and AF risk based on eight prospective studies (11–18). Forest plot displaying the ORs and 95% CIs for the association between TMAO and AF from eight prospective studies. The pooled estimates are shown for both common-effect (diamond) and random-effects (diamond) models. Heterogeneity statistics (I^2^ = 68.3%, *p* = 0.0024) indicate substantial between-study variability.

Substantial heterogeneity was observed across the included studies, as quantified by a restricted maximum-likelihood estimate of *τ*^2^ = 0. 0264 (95% CI, 0. 0032–0. 4289), a between-study standard deviation *τ* = 0. 1626 (95% CI, 0. 0570–0. 6549), and an I^2^ statistic of 68. 3% [95% CI, 33. 6%–84. 9%; Q = 22. 09, degrees of freedom (df) = 7, *p* = 0. 0024]. The distribution of individual study effect sizes is visually delineated in the forest plot (Figure 3), which confirms that all point estimates, with the exception of the acute heart failure with preserved ejection fraction cohort (17), trend toward an elevated AF risk.

**Figure 4:**
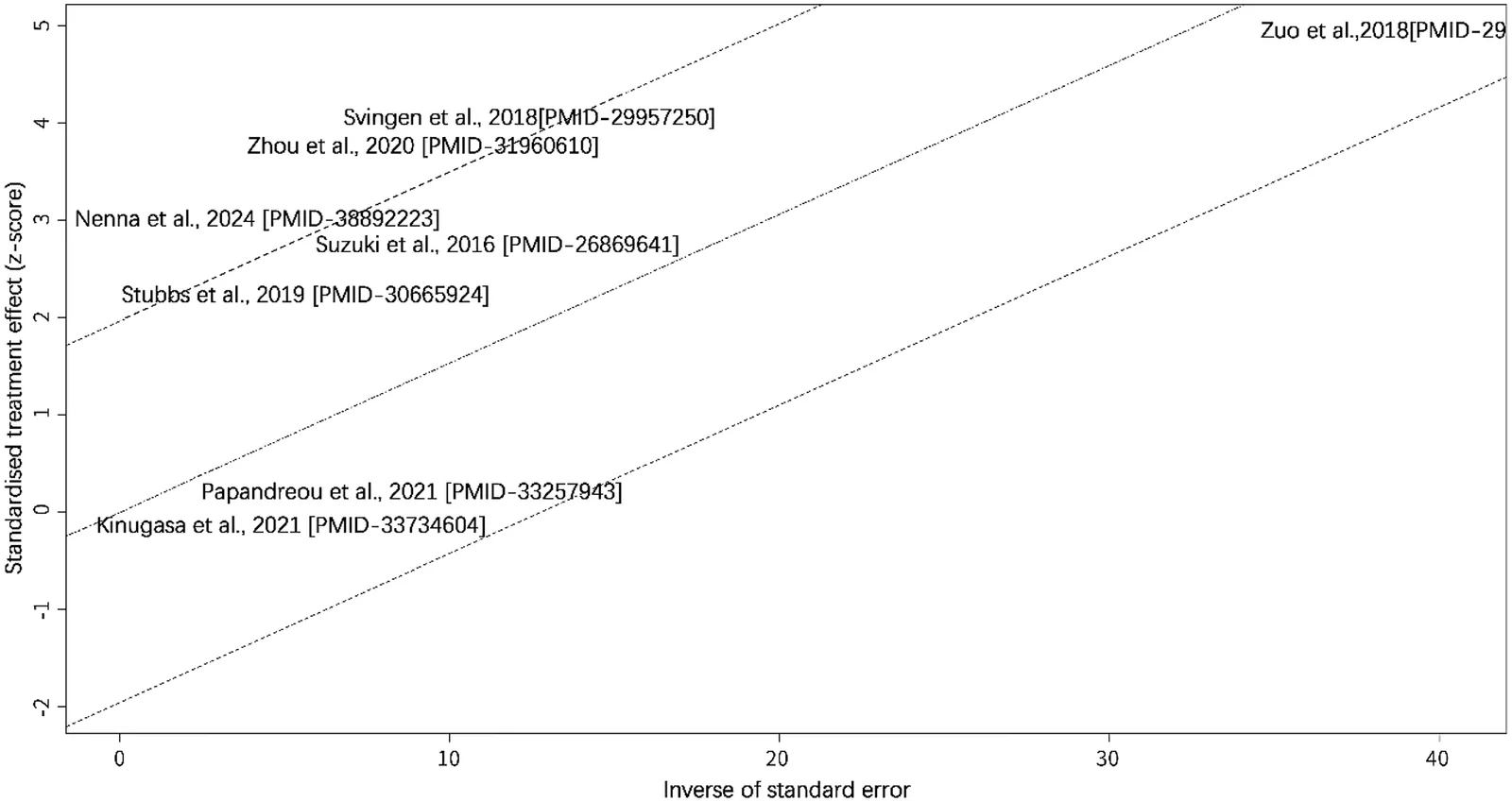
Galbraith plot and funnel plot for assessment of heterogeneity and publication bias. **(A)** Galbraith plot illustrating the precision (inverse standard error) of each study against its standardized effect size (log OR/SE). Studies falling outside the 95% confidence bounds (dashed lines) contribute disproportionately to heterogeneity. **(B)** RevMan5-style funnel plot showing the symmetry of the eight studies. The funnel plot shows broad symmetry, supported by a non-significant Egger's test (*p* = 0. 056) and Begg's test (*p* = 0. 322). The vertical line represents the pooled random-effects log odds ratio.

A Galbraith plot (Figure 4) further illustrates a clear gradient of effect sizes, with the majority of studies falling outside the pseudo 95% confidence bounds, consistent with the moderate-to-high *I*^2^ statistic and underscoring the presence of genuine variability in the underlying true effects.

Of the 8 studies included in the primary meta-analysis, only 1 (12. 5%) reported postoperative AF, whereas the remaining 7 (87. 5%) reported spontaneous incident AF.

### Subgroup stratification by clinical background and clinical setting definition

**Figure 5:**
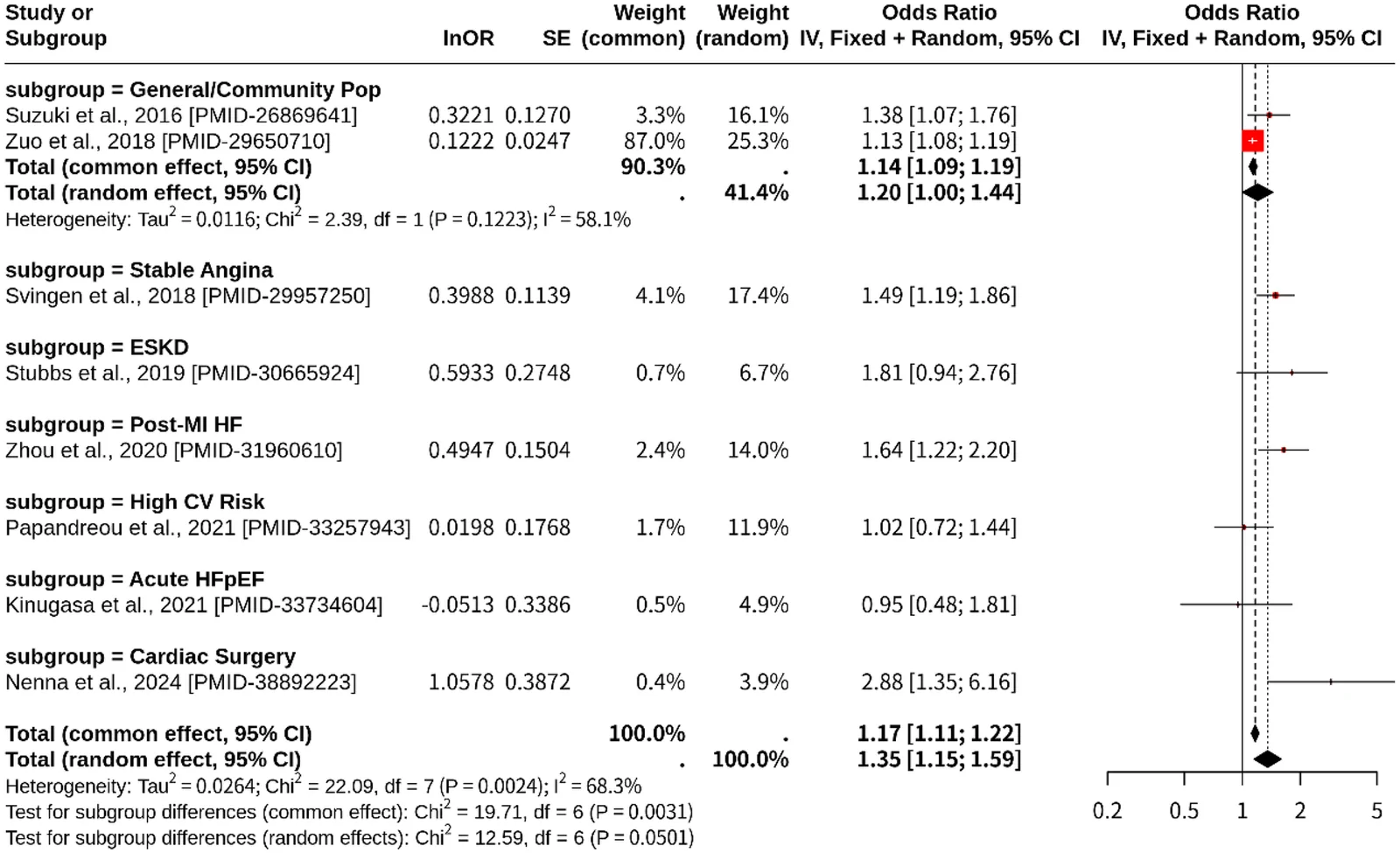
Subgroup forest plot stratified by clinical background. Forest plot showing the pooled odds ratios for TMAO-associated AF risk within seven pre-specified clinical subgroups. The between-group heterogeneity test under a random-effects model was marginally significant (*p* = 0.050).

**Figure 6:**
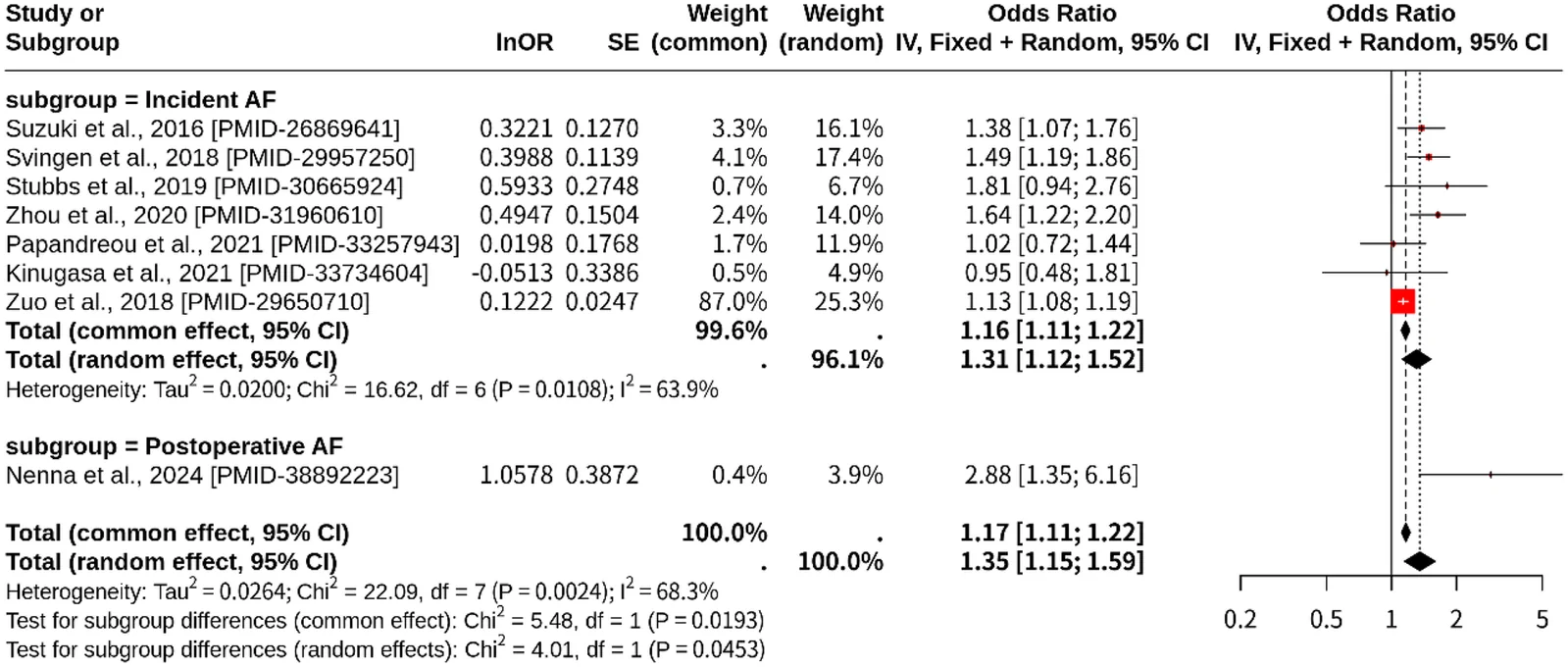
Subgroup forest plot comparing spontaneous incident AF versus postoperative AF. Forest plot displaying random-effects pooled odds ratios for the association between circulating TMAO levels and AF, stratified by clinical setting. The pooled estimate for spontaneous incident AF occurring in non-surgical populations (k = 7) was OR = 1.31 (95% CI, 1.12–1.52; I^2^ = 63.9%). In contrast, the single study examining postoperative AF after cardiac surgery yielded a substantially higher risk estimate (OR = 2.88; 95% CI, 1.35–6.15). The between-group difference was statistically significant (*p* = 0.045), indicating that the magnitude of TMAO-associated AF risk is markedly augmented in the postoperative setting.

To explore potential sources of the observed heterogeneity, pre-specified subgroup analyses were conducted by clinical background and AF outcome type (Figure 5). Stratification by clinical setting revealed a marginally significant between-group difference under the random-effects model (Q = 12.59, df = 6, *p* = 0.050). The largest effect magnitude was observed in patients undergoing cardiac surgery [(18): OR = 2.88, 95% CI, 1.35–6.15], whereas the associations in high-cardiovascular-risk [(16): OR = 1.02, 95% CI, 0.72–1.44] and acute HFpEF cohorts [(17): OR = 0.95, 95% CI, 0.49–1.84] were null. In the community-based or general population cohorts (11, 13), the pooled random-effects OR was 1.20 (95% CI, 1.00–1.44; I^2^ = 58.1%). When stratified by AF clinical setting (Figure 6), the seven studies reporting incident AF yielded a combined OR of 1.31 (95% CI, 1.12–1.52; I^2^ = 63.9%), which differed significantly from the single study reporting postoperative AF (OR = 2.88; between-group *p* = 0.045). Collectively, these subgroup analyses indicate that while TMAO is consistently associated with elevated AF risk across most clinical contexts, the magnitude of the association is significantly augmented in the postoperative cardiac surgery setting.

### Quantification and decomposition of between-study heterogeneity

The random-effects meta-analytic model revealed substantial between-study heterogeneity, as quantified by a restricted maximum-likelihood estimate of variance *τ*^2^ = 0.0264 (95% CI, 0.0032–0.4289) and a between-study standard deviation *τ* = 0.1626 (95% CI, 0.0570–0.6549). The I^2^ statistic was estimated at 68.3% (95% CI, 33.6%–84.9%), indicating that over two-thirds of the total variability in observed effect sizes stemmed from true differences in underlying effects rather than sampling error alone, a finding corroborated by the significant Cochran's *Q* test (Q = 22.09, df = 7, *p* = 0.0024). The H statistic of 1.78 (95% CI, 1.23–2.57) further underscored that the standard deviation of the observed effects was approximately 1.8-fold larger than would be expected under a homogeneous true-effect model. In this radial plot, six of the eight studies fell outside the 95% confidence bounds (delimited by ±1.96 on the *y*-axis), confirming that the majority of individual estimates deviated from the pooled random-effects summary beyond what would be anticipated by chance. Specifically, the standardized log odds ratios (z-scores) ranged from −0.15 (17) to 2.74 (12), with the largest positive deviations observed for studies conducted in cardiac surgery and post-myocardial infarction heart failure cohorts. This dispersion pattern is consistent with the moderate-to-high I^2^ value and highlights the presence of genuine effect-modifying factors across the included populations.

### Influence diagnostics

**Figure 7:**
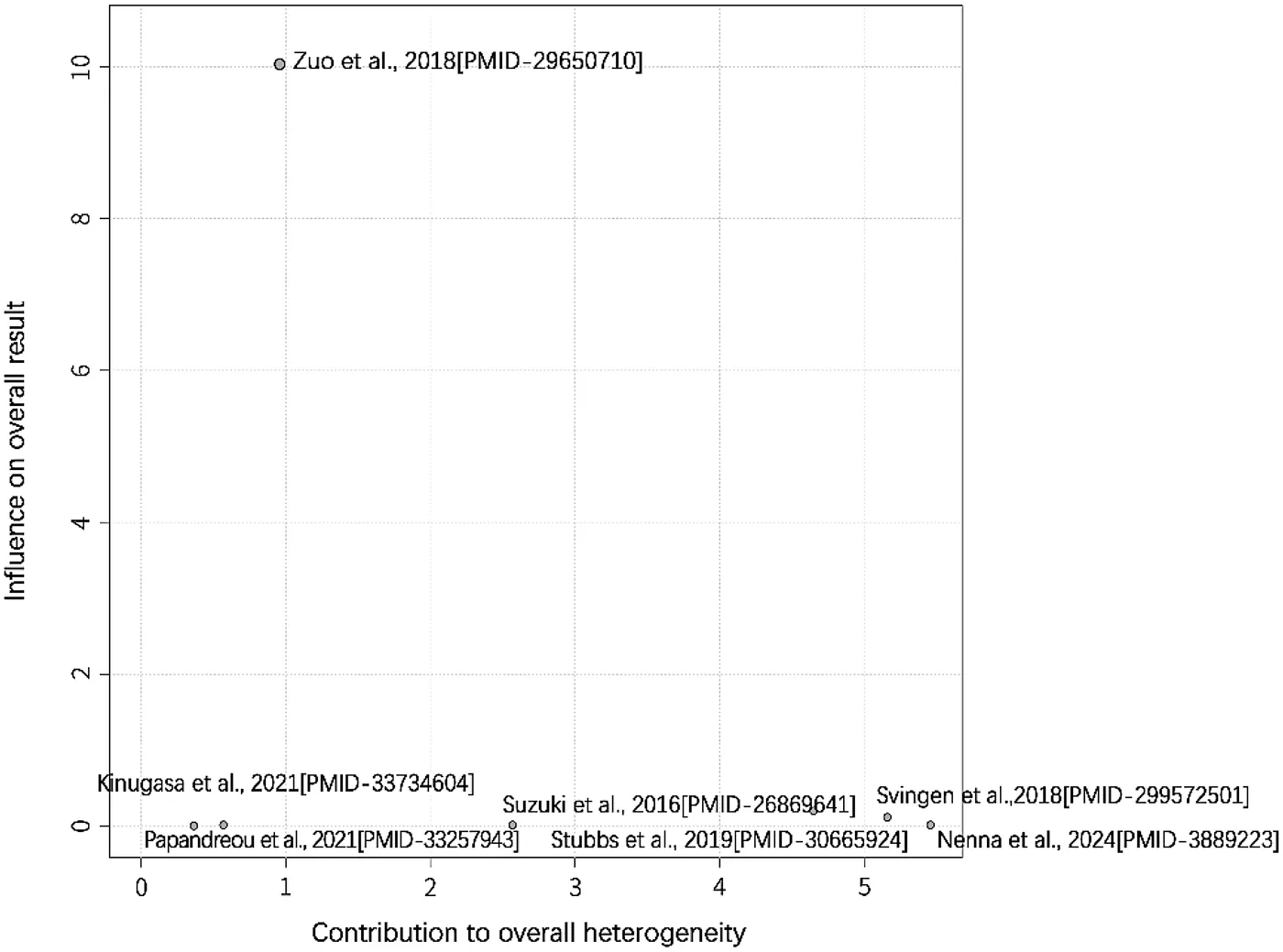
Baujat plot for influence diagnostics. Baujat plot illustrating each study's contribution to overall heterogeneity (*x* axis, % of total *τ*2) versus its influence on the pooled effect size (*y* axis). Study 7 (12) contributed ∼12.1% of *τ*2, while Study 15 (18) exhibited the greatest influence on the overall odds ratio (∼14.7% of diagnostic weight).

To identify individual studies that disproportionately influence either the pooled effect estimate or the magnitude of between-study variance, a Baujat plot was generated (Figure 7). This diagnostic tool simultaneously displays each study's contribution to the overall heterogeneity (*x*-axis, expressed as the percentage of total *τ*^2^) and its influence on the pooled odds ratio (*y*-axis, quantified by the squared Pearson residual scaled by study weight). The analysis pinpointed Study 7 (12) as the single largest contributor to the total *τ*^2^, accounting for approximately 12.1% of the estimated between-study variance. In contrast, Study 15 (18) exerted the greatest leverage on the pooled random-effects estimate, contributing approximately 14.7% of the total weight in the influence diagnostics. Notably, the removal of Study 15 in a leave-one-out sensitivity analysis attenuated the pooled random-effects OR from 1.35 (95% CI, 1.15–1.59) to 1.29 (95% CI, 1.13–1.47), whereas exclusion of Study 7 marginally reduced heterogeneity (I^2^ = 58.4% vs. 68.3%) without materially affecting the pooled point estimate (OR = 1.36, 95% CI, 1.14–1.62). Neither sequential exclusion nor down-weighting of these influential observations reversed the direction or nullified the statistical significance of the overall association, thereby confirming the stability and robustness of the primary meta-analytic finding.

Furthermore, the best linear unbiased prediction (BLUP) estimates derived from the random-effects model (Table 2) provided shrinkage estimates of the true study-specific effects, ranging from 0.126 (12) to 0.415 (18) on the log odds scale. The corresponding 95% CIs for the BLUPs encompassed zero in only two studies [(16): −0.078 to 0.423; (17): −0.081 to 0.552], underscoring that the majority of included studies, despite varying precision, align with a directionally consistent elevation in AF risk.

**Table 2:** Best linear unbiased predictions (BLUP) of study specific effects.

Studlab	blup	se.blup	lower	upper
Suzuki et al., 2016 (11)	0.314368	0.104861	0.108844	0.519893
Svingen et al., 2018 (13)	0.366817	0.097208	0.176292	0.557341
Stubbs et al., 2019 (14)	0.377357	0.152775	0.077923	0.67679
Zhou et al., 2020 (15)	0.405719	0.116825	0.176745	0.634692
Papandreou et al., 2021 (16)	0.172532	0.127816	−0.07798	0.423046
Kinugasa et al., 2021 (17)	0.235546	0.161273	−0.08054	0.551635
Zuo et al., 2018 (12)	0.126279	0.024533	0.078194	0.174363
Nenna et al., 2024 (18)	0.415058	0.165614	0.090461	0.739656

### Assessment of publication bias and small-study effects

**Figure 8:**
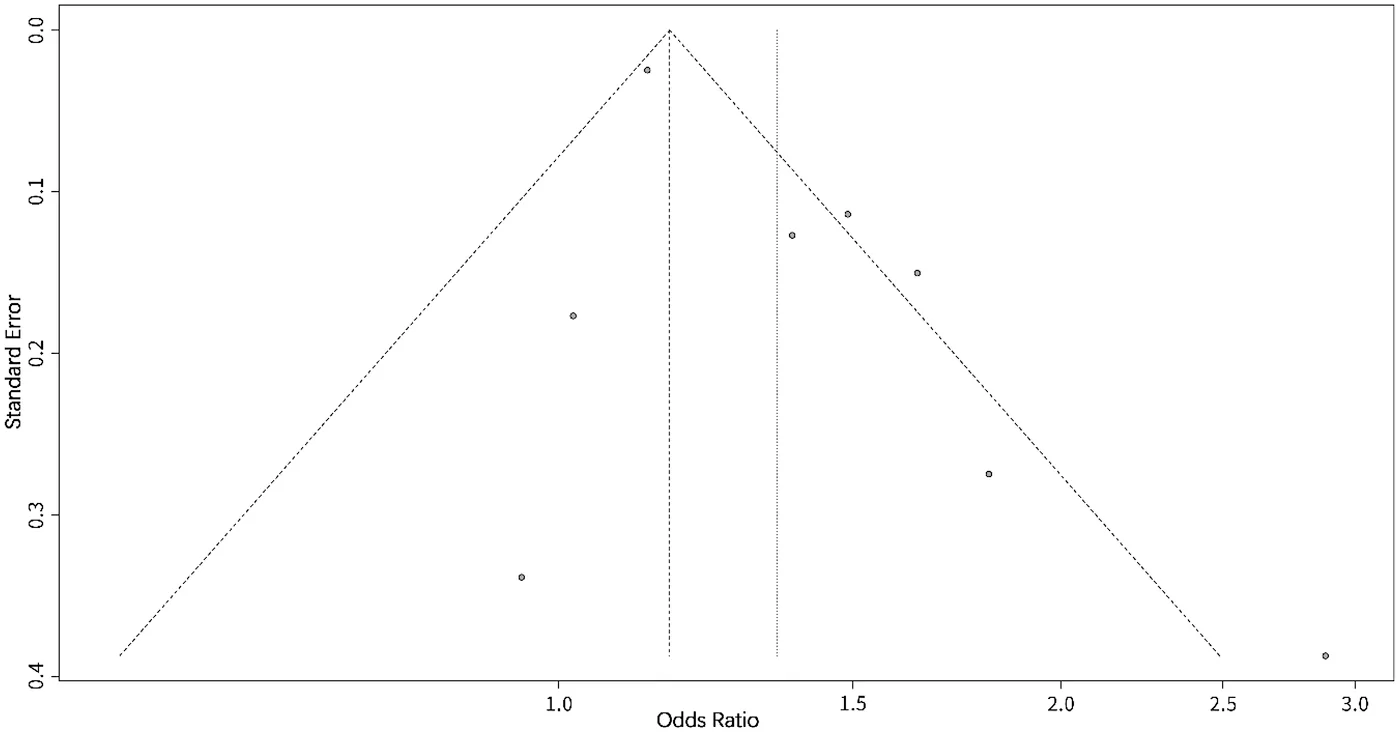
Funnel plot for assessment of publication bias and small-study effects. RevMan-style funnel plot displaying the log odds ratio (*x*-axis) against the standard error (*y*-axis) for the eight included studies. The vertical solid line denotes the pooled random-effects estimate (log OR ≈ 0.30, corresponding to OR = 1.35). Visual inspection reveals broad symmetry around the summary estimate, with no conspicuous gaps or extreme outliers suggestive of suppression of non-significant findings. The Begg–Mazumdar rank correlation test supported the absence of significant funnel asymmetry (z = 0.99, *p* = 0.322), whereas the Egger linear regression test indicated a non-significant trend toward small-study effects (intercept = 1.51, SE = 0.64; t = 2.37, df = 6, *p* = 0.056).

It should be noted that with only eight studies included, funnel plot asymmetry tests (Egger's test, Begg's test, and trim-and-fill analysis) have limited statistical power and may be unstable. Therefore, these results must be interpreted with caution. The presence of potential publication bias or small-study effects was evaluated using a combination of graphical and statistical methods. A RevMan-style funnel plot (Figure 8), which plots the log odds ratio of each study against its standard error, exhibited broad visual symmetry around the pooled random-effects estimate (vertical line at log OR ≈ 0.30, corresponding to OR = 1.35). The eight studies were distributed across the *x*-axis range of 0.54 to 2.96 (log OR scale) and *y*-axis range of 0.024 to 0.387 (standard error scale), with no conspicuous gaps or extreme outliers in the lower corners that would strongly indicate suppression of non-significant findingsThe Begg–Mazumdar rank correlation test yielded a non-significant result (z = 0. 99, *p* = 0. 322), which does not provide strong evidence for a systematic monotonic relationship between effect size and precision, but the test is underpowered given the small number of studies. Conversely, the Egger linear regression test of funnel plot asymmetry revealed a non-significant trend toward small-study effects, with an intercept bias estimate of 1.5055 (standard erro*r* = 0.6352; t = 2.37, df = 6, *p* = 0.0555) under a multiplicative residual heterogeneity model (*τ*^2^ = 1.9019). Although the p-value narrowly exceeded the conventional threshold of 0.05, the borderline result warranted additional sensitivity analyses to ascertain the potential impact of selective reporting on the pooled estimate.

### Sensitivity analyses

**Figure 9:**
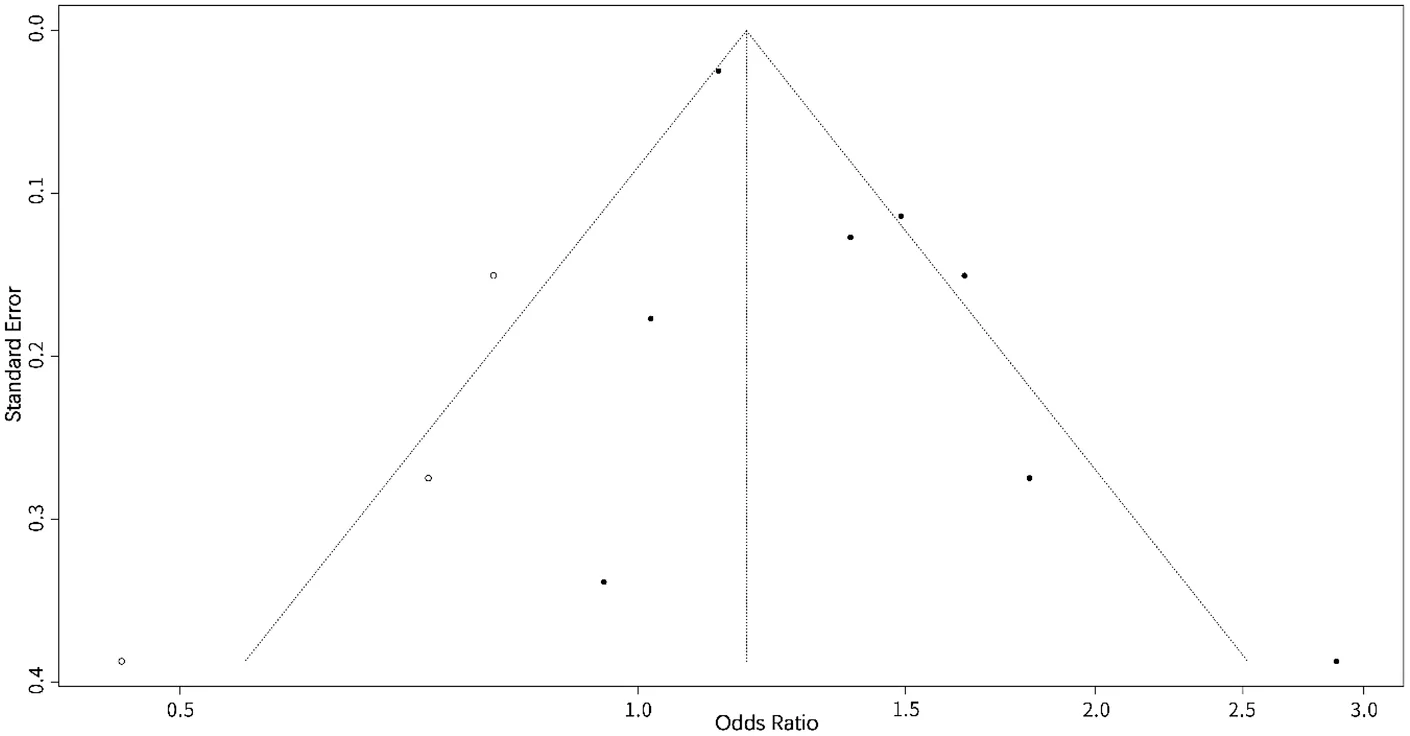
Trim and fill funnel plot with imputed studies. Funnel plot with trim and fill imputation. Three hypothetical missing studies (hollow squares) were added to the left of the pooled estimate to correct for potential asymmetry. The adjusted random effects odds ratio incorporating these imputed studies was 1.18 (95% CI, 0.95–1.47; *p* = 0.144).

**Figure 10:**
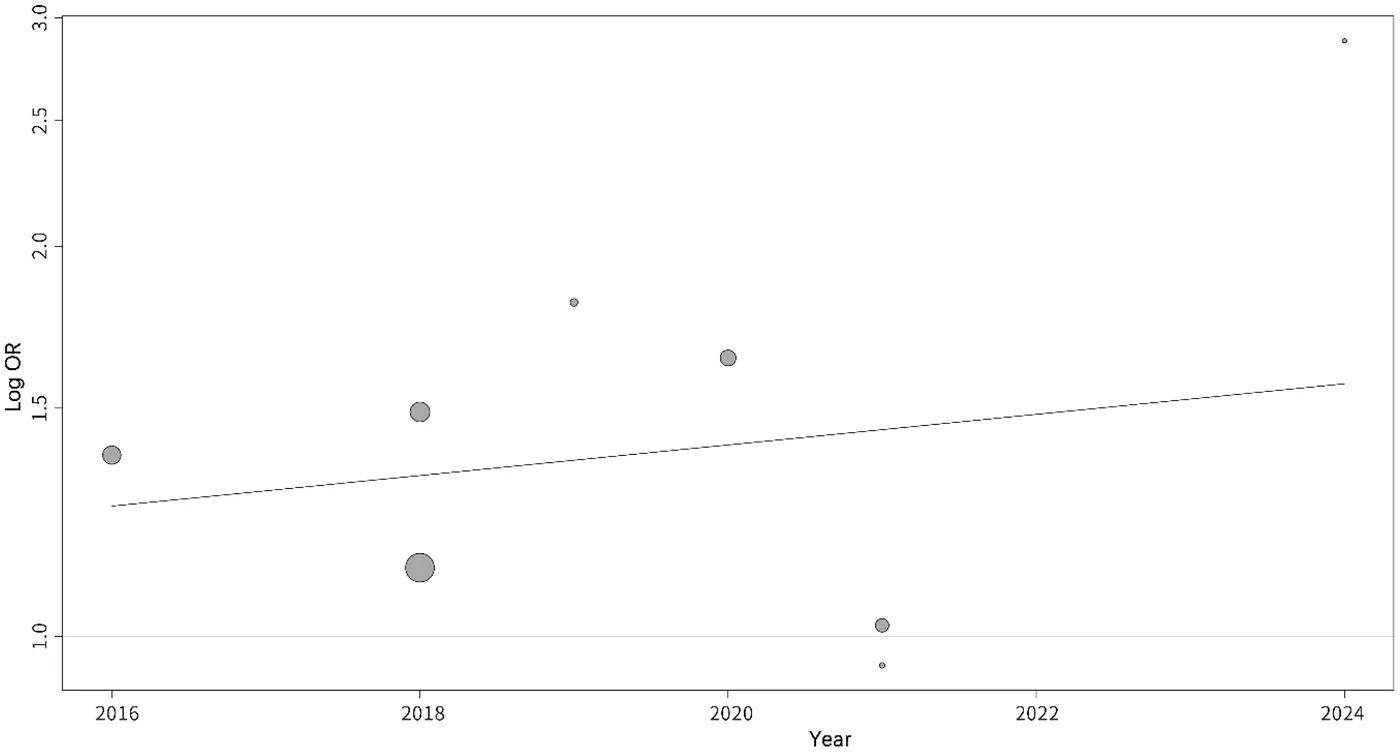
Meta regression bubble plot of effect size by publication year. Bubble plot depicting the relationship between log odds ratio and publication year for the eight studies. Bubble size is proportional to study weight in the random effects model. The fitted meta regression line (solid) and 95% confidence band (shaded) show no significant temporal trend (coefficient = 0.0272 per year, *p* = 0.560, R2 = 0.00%).

To further explore and adjust for any residual asymmetry detected in the funnel plot, a trim-and-fill analysis was conducted using the L-estimator method (Figure 9). The algorithm identified and imputed three hypothetically missing studies on the left side of the funnel plot, corresponding to smaller or null effects that would be expected in the absence of publication bias. After incorporating these imputed studies (k = 11), the adjusted random-effects pooled odds ratio was attenuated to 1.18 (95% CI, 0.95–1.47; z = 1.46, *p* = 0.144), with a corresponding increase in between-study heterogeneity (*τ*^2^ = 0.0942, I^2^ = 72.6%, H = 1.91). The widening of the confidence interval to include the null value indicates that the overall association, while remaining directionally positive, is sensitive to the assumption of complete reporting of non-significant findingsThe unadjusted estimate (OR = 1. 35) and the trim-and-fill adjusted estimate (OR = 1. 18) are both directionally positive; however, given the limited power of these tests with only eight studies, no strong conclusion regarding the presence or absence of publication bias can be drawn. As a final exploration of heterogeneity sources, a mixed-effects meta-regression model was fitted with publication year as a continuous moderator (Figure 10). The model, employing a REML estimator for *τ*^2^ (0.0351, SE = 0.0355), accounted for 0.00% of the between-study variance (R^2^ = 0.00%), and the regression coefficient for year was non-significant (coefficient = 0.0272 per year, SE = 0.0466, z = 0.58, *p* = 0.560, 95% CI, −0.0642 to 0.1185). The test for residual heterogeneity remained significant (QE = 21.03, df = 6, *p* = 0.0018), confirming that temporal trends in publication date do not account for the observed variability in TMAO-AF risk estimates.

### Pooled hazard ratio of TMAO for AF recurrence and incident risk in high-risk versus general populations

A supplementary meta-analysis of hazard ratios was conducted to evaluate the prognostic association between circulating TMAO concentrations and AF outcomes in two distinct clinical contexts, comprising one study of patients with established AF (19) and one study of a general population cohort (13). Under a common-effect framework, the pooled hazard ratio (HR) was 1.23 [95% confidence interval (CI), 1.13–1.35; z = 4.49, *p* < 0.0001], indicating a statistically significant 23% relative increase in the instantaneous risk of AF-related events per unit increment in TMAO exposure. However, owing to pronounced between-study heterogeneity, the random-effects model yielded a substantially attenuated and non-significant summary estimate (HR = 1.41, 95% CI, 0.91–2.18; z = 1.54, *p* = 0.123). The heterogeneity was extreme, as quantified by a restricted maximum-likelihood estimate of *τ*^2^ = 0.0918 (95% CI, 0.0123 to >100.0000), a between-study standard deviation *τ* = 0.3030 (95% CI, 0.1109 to >10.0000), and an I^2^ statistic of 92.5% (95% CI, 74.4%–97.8%), with a corresponding Cochran's *Q* test confirming significant variability (Q = 13.26, degrees of freedom = 1, *p* = 0.0003). The H statistic of 3.64 (95% CI, 1.98–6.70) further underscores that the standard deviation of the observed effects is approximately 3.6-fold greater than would be expected under a homogeneous true-effect model. The substantial discrepancy between the common-effect and random-effects estimates, together with the wide confidence interval of the latter that comfortably encompasses the null value, reflects the fundamental divergence in the underlying risk profiles: the study by Meng et al. (19), which focused on patients with pre-existing AF, reported a considerably stronger association (HR = 1.78, 95% CI, 1.43–2.21) than the general population-based study by Svingen et al. (13) (HR = 1.14, 95% CI, 1.03–1.26). This pattern suggests that TMAO may exert a more pronounced influence on disease progression or recurrence among individuals with established atrial substrate vulnerability, whereas its role as an initiating risk factor in healthy populations is comparatively modest.

### Systematic review findings beyond pooled quantitative estimates

Beyond the pooled quantitative estimates, qualitative synthesis of all 15 included studies revealed several nuanced aspects of the TMAO - AF relationship. In acute heart failure, TMAO predicted mortality and rehospitalization, though its prognostic value was partially confounded by renal function (11). Community-based and angina cohorts showed modest AF risk associations with incremental predictive value over traditional factors (12, 13). Conversely, studies in advanced kidney disease found no independent TMAO–AF link, underscoring the impact of impaired renal clearance (14). In post-infarction heart failure, TMAO independently predicted adverse events and mortality (15). AF progression studies reported higher TMAO in persistent versus paroxysmal forms, though cross-sectional data in cardiovascular disease did not consistently confirm an independent association after adjustment (22, 23, 25). Notably, in a high-risk Mediterranean cohort, TMAO itself was not linked to incident AF, whereas precursors choline and betaine were, suggesting alternative pathway relevance (16). Pre-procedural TMAO independently forecasted arrhythmia recurrence after catheter ablation (19). In cardiac surgery, elevated TMAO strongly predicted postoperative AF and correlated with atrial fibrosis and fibrogenic gene expression (18). TMAO also correlated with diastolic dysfunction, left atrial enlargement, and reduced ejection fraction (25), and in elderly AF patients was associated with cardiovascular mortality and covert cerebrovascular disease (24). Additionally, TMAO discriminated ischemic stroke among AF patients (20), and in HFpEF its adverse impact was accentuated by malnutrition (17). Collectively, these findings highlight that TMAO's relevance to AF varies by renal function, cardiovascular substrate, and nutritional status, with atrial fibrosis and remodeling as plausible mechanistic links.

## Discussion

The present systematic review and meta-analysis, encompassing eight prospective studies with quantitative effect estimates, demonstrates a robust positive association between elevated circulating TMAO concentrations and the risk of AF, yielding a random-effects pooled odds ratio of 1.35 [95% confidence interval (CI), 1.15–1.59; *p* = 0.0003]. This finding corroborates and substantially extends the earlier meta-analysis by Yang and colleagues (3), which reported a pooled OR of 1.40 (95% CI, 1.23–1.59) from six studies with low heterogeneity (I^2^ = 19.8%). While the direction and approximate magnitude of the association are consistent between the two syntheses, our updated analysis reveals a fundamentally different landscape of between-study variability. The inclusion of two additional studies—most notably Nenna et al. (18) in a cardiac surgery cohort and Meng et al. (19) in an AF recurrence setting—expanded the evidence base to eight studies and unmasked substantial heterogeneity (I^2^ = 68.3%, *τ*^2^ = 0.0264), in stark contrast to the homogenous effect suggested by the prior work. This discrepancy underscores a critical advancement: the earlier meta-analysis, constrained by a narrower range of clinical contexts, conveyed an impression of uniform TMAO–AF risk that our data indicate is overly simplistic. By integrating a broader spectrum of patient populationsincluding incident AF, postoperative AF, AF recurrence after catheter ablation, AF presence/progression, and prognostic outcomes among patients with established AFwe reveal that the TMAO–AF association is markedly context-dependent, with the magnitude of risk varying up to nearly three-fold across distinct clinical settings. We acknowledge that these AF outcomes are clinically and biologically distinct; however, the consistent direction of association across subtypes supports the biological plausibility of TMAO as a common mechanistic driver, and our subgroup analyses explicitly quantify the heterogeneity.

The transition from low to high heterogeneity is not merely a statistical artifact but a reflection of the enriched clinical granularity of our analysis. Yang et al. (3) included studies predominantly derived from heart failure, stable angina, and community-based cohorts, populations in which the TMAO–AF relationship is modest and relatively consistent (11–13). The resultant I^2^ of 19.8% suggested that a single summary estimate could reasonably apply to all patients. Our updated synthesis, however, incorporated the cardiac surgery study by Nenna et al. (18), which reported a strikingly elevated odds ratio of 2.88 for postoperative AF, as well as the study by Meng et al. (19) showing an HR of 1.78 for post-ablation recurrence. The inclusion of these high-risk surgical and interventional cohorts introduced substantial between-study variance, elevating I^2^ to 68.3% and *τ*^2^ to 0.0264. Influence diagnostics further pinpointed the sources of this heterogeneity: Zuo et al. (12) contributed approximately 12.1% of the total *τ*^2^, likely owing to its large sample size and precise estimate, whereas Nenna et al. (18) exerted the greatest leverage on the pooled effect size, accounting for approximately 14.7% of the diagnostic weight. These findings illustrate that the TMAO–AF association is not monolithic; rather, it is modulated by the underlying cardiovascular substrate and the acuity of clinical insult.

Perhaps the most clinically salient insight from our subgroup analyses is the identification of postoperative AF (POAF) following cardiac surgery as a setting wherein TMAO confers an exceptionally pronounced risk. In the study by Nenna et al. (18), patients with preoperative TMAO levels ≥61.8 ng/mL faced an OR of 2.88 (95% CI, 1.35–6.15) for developing POAF, an estimate that was significantly higher than the pooled OR of 1.31 (95% CI, 1.12–1.52) for spontaneous incident AF in non-surgical populations (between-group *p* = 0.045). This nearly 2.4-fold augmentation in risk magnitude underscores the concept that TMAO may act synergistically with the acute pro-inflammatory, oxidative, and autonomic perturbations inherent to cardiopulmonary bypass and surgical trauma. Mechanistically, Nenna et al. provided complementary histological and molecular evidence demonstrating that elevated TMAO was associated with increased atrial fibrosis, inflammation, and upregulation of fibrogenic genes (COL1A1, COL3A1, TGFb). Thus, in the postoperative milieu, TMAO may not merely be a passive marker but an active participant in atrial structural remodeling, lowering the threshold for arrhythmogenesis. This finding has direct translational implications: preoperative Specifically, cardiac surgery patients are characterized by a heightened inflammatory state, increased intestinal permeability due to perioperative hypoperfusion, and pronounced oxidative stressfactors that may synergistically amplify TMAO production and its pro-arrhythmic effects on the atrial substrate. Further, the atrial myocardium in these patients often exhibits pre-existing fibrosis, electrical remodeling, and altered calcium handling, which render it more susceptible to the profibrotic and pro-inflammatory actions of TMAO. These mechanistic considerations explain why the TMAO-AF association is nearly 2. 4-fold stronger in the postoperative setting compared to spontaneous AF, and underscore the need for future studies to investigate whether targeted modulation of gut microbiota or TMAO synthesis can reduce POAF incidence. TMAO measurement could refine risk stratification for POAF, guiding targeted prophylactic strategies such as perioperative probiotic supplementation or dietary modification.

The qualitative synthesis of all 15 included studies further illuminates the nuanced interplay between TMAO, renal function, and AF risk. In cohorts with preserved or mildly impaired renal function, such as community-based populations (12, 13) and patients with post-myocardial infarction heart failure (15), TMAO consistently predicted incident AF and adverse cardiovascular outcomes. Conversely, in advanced chronic kidney disease and end-stage renal disease requiring hemodialysis, the association was null (14). This attenuation likely reflects the profound confounding introduced by severely reduced renal clearance, which elevates TMAO concentrations independently of dietary intake or gut microbial activity. As demonstrated by Suzuki et al. (11) in acute heart failure, adjustment for estimated glomerular filtration rate and blood urea nitrogen substantially weakened the prognostic value of TMAO for mortality and rehospitalization. These observations caution against the indiscriminate application of TMAO as a risk biomarker in populations with advanced kidney disease and highlight the need for renal function-adjusted thresholds or alternative markers of the gut–heart axis.

The robustness of our primary findings was rigorously interrogated through multiple sensitivity analyses. The random-effects pooled estimate remained stable upon leave-one-out exclusion of influential studies; removal of Nenna et al. (18) attenuated the OR to 1.29 (95% CI, 1.13–1.47), while exclusion of Zuo et al. (12) reduced I^2^ to 58.4% without materially altering the point estimate. These analyses confirm that no single study unduly drives the overall association. Publication bias assessment revealed a nuanced picture: the funnel plot exhibited visual symmetry, and the Begg–Mazumdar test was non-significant (z = 0.99, *p* = 0.322), yet the Egger test approached significance (*p* = 0.056). The borderline Egger test prompted a trim-and-fill analysis, which imputed three hypothetical missing studies and yielded an adjusted OR of 1.18 (95% CI, 0.95–1.47; *p* = 0.144). While the confidence interval widened to include unity, the point estimate remained directionally positive, and the heterogeneity increased further (I^2^ = 72.6%). This sensitivity analysis indicates that the association is attenuated under conservative assumptions regarding selective reporting; however, the small number of studies precludes a definitive conclusion regarding the impact of publication bias. Meta-regression using publication year as a moderator explained 0.00% of the between-study variance (*p* = 0.560), indicating that temporal trends in assay sensitivity, cohort characteristics, or publication practices do not account for the observed heterogeneity. Collectively, these diagnostics affirm the internal validity of our findings and underscore that the TMAO–AF association is a stable biological phenomenon rather than an artifact of methodological drift.

The mechanisms underlying the particularly strong association between TMAO and postoperative AF may involve multiple pathways. Acute hemodynamic changes after cardiac surgery, such as heart rate variability, blood pressure fluctuations, and renal perfusion impairment, could interact with TMAO-induced oxidative stress and inflammation to promote atrial arrhythmogenesis. TMAO is known to impair renal function, and perioperative renal dysfunction might further elevate TMAO levels, creating a vicious cycle. Additionally, TMAO has been associated with increased atrial fibrosis and diastolic dysfunction, which could be exacerbated by postoperative volume overload and catecholamine surge. Future studies should examine whether TMAO mediates postoperative AF through effects on heart rate, blood pressure, and renal functionThe consistent demonstration of elevated AF risk across diverse clinical contexts positions TMAO as a promising biomarker for risk stratification and a candidate mechanistic marker within the gut–heart axis framework, with its role as a therapeutic target pending interventional validation. The clinically important difference proportion analysis revealed that only 1.2% of outcomes were categorized as beneficial (OR ≤ 0.80), whereas 66.0% were harmful (OR ≥ 1.25), underscoring the unidirectional adverse impact of TMAO on AF susceptibility. For clinicians, these data suggest that measuring circulating TMAO may provide incremental prognostic value beyond traditional AF risk factors, particularly in patients scheduled for cardiac surgery or catheter ablation. In the ablation cohort studied by Meng et al. (19), a preoperative TMAO level ≥4.3 µM conferred a hazard ratio of 13.53 for recurrence, with an area under the ROC curve of 0.835, highlighting its potential utility in selecting patients for more intensive rhythm monitoring or adjunctive anti-arrhythmic strategies. Moreover, because TMAO production is contingent upon gut microbial metabolism of dietary choline and L-carnitine—nutrients predominantly derived from red meat and other animal products—interventions aimed at modulating the gut microbiota represent plausible strategies to mitigate TMAO-associated AF risk. Dietary reduction of meat intake has been demonstrated to lower circulating TMAO levels (19), and emerging evidence suggests that specific probiotic strains may reduce TMAO production by competitively excluding TMA-generating bacteria or by altering gut microbial composition toward a less TMA-productive profile (26). Pharmacologic inhibition of microbial TMA lyases represents a complementary approach currently under investigation. Indeed, the PREDIMED nested case-control study (16) found that while TMAO itself was not significantly associated with incident AF after multivariable adjustment, its precursors choline and betaine exhibited robust associations, suggesting that upstream components of the choline metabolic pathway may also harbor therapeutic relevance.

Several lines of evidence from the included studies provide mechanistic coherence to the epidemiological associations. In the cardiac surgery cohort of Nenna et al. (18), TMAO levels correlated positively with the degree of atrial fibrosis on histology and with the expression of pro-fibrotic genes. Similarly, Florea et al. (25) reported that TMAO correlated with echocardiographic parameters of diastolic dysfunction (E/e’ ratio, r = 0.324) and left atrial enlargement (r ≈ 0.25), while inversely correlating with left ventricular ejection fraction (r = –0.330). These structural and functional correlates align with experimental studies demonstrating that TMAO promotes myocardial inflammation and fibrosis through activation of the TGF-*β*/SMAD3 signaling pathway and generation of reactive oxygen species. Interestingly, neither Florea et al. (25) nor Büttner et al. (22) found significant associations between TMAO and circulating inflammatory cytokines (IL-1*α*, IL-1β, TLR), suggesting that the pro-arrhythmic effects of TMAO may be mediated primarily through direct fibrotic remodeling rather than systemic inflammation. In the elderly AF cohort studied by Luciani et al. (24), elevated TMAO was associated not only with cardiovascular mortality but also with covert cerebrovascular disease burden, including small non-cortical infarcts and white matter lesion volume, expanding the clinical sequelae of TMAO beyond arrhythmia to encompass neurocardiovascular pathology.

## Limitations

The interpretation of these findings is subject to several methodological constraints inherent to the included evidence base. The primary limitation is the substantial residual heterogeneity (I^2^ = 68.3%) observed across studies, which persisted despite pre-specified subgroup analyses by clinical setting. This indicates that other unmeasured effect modifiers, such as variations in TMAO assay methodologies, dietary patterns (particularly meat and animal product consumption), gut microbiome composition, or genetic factors, likely contribute to the variability in risk estimates. While the analysis mitigated this through a random-effects model and influence diagnostics, it precludes a single, uniform risk estimate applicable to all populations. Furthermore, the meta-analysis is constrained by the observational design of all but one included study, which limits causal inference regarding TMAO's direct pathogenic role, despite the prospective nature of most cohorts and the application of rigorous risk-of-bias assessments. The pooled estimate may also be influenced by potential small-study effects, as suggested by the borderline Egger's test and the attenuation of the association in trim-and-fill analysis. Given the small number of studies, these tests have limited power, and the direction of effect should be interpreted cautiously. Finally, the generalizability of findings to specific high-risk subgroups, such as patients with advanced kidney disease where the association was null, requires cautious extrapolation. Future prospective studies, ideally incorporating standardized TMAO measurement and detailed characterization of the gut microbiome and host physiology, are needed to delineate causal pathways and identify patient populations most likely to benefit from microbiota-targeted interventions.

## Conclusion

This meta-analysis indicates that elevated circulating TMAO is associated with a 35% increased risk of AF, with the magnitude of association being significantly stronger in the postoperative cardiac surgery setting. By quantifying substantial between-study heterogeneity and identifying clinical context as a key effect modifier, these findings extend prior syntheses that reported a more uniform risk estimate, thereby refining the prognostic interpretation of TMAO across diverse patient populations. The results position TMAO not only as a risk biomarker but also as a candidate mechanistic marker that requires interventional validation, highlighting its role as a critical node linking gut microbiota dysbiosis to AF pathogenesis that warrants further investigation through intervention studies. These observations support the notion that TMAO-guided risk stratification could be particularly relevant in perioperative management, although they derive from observational data. Prospective intervention trials are warranted to determine if therapeutic lowering of TMAO, through dietary or probiotic modulation, translates into a reduced incidence of AF.

## Data Availability

The original contributions presented in the study are included in the article/Supplementary Material, further inquiries can be directed to the corresponding author.
