# Supplementary material for: Gut microbiota-derived trimethylamine N-oxide and the incidence and recurrence of atrial fibrillation: a systematic review and meta-analysis
Source: Front Cardiovasc Med. 2026 Aug 24;13:1897722. doi: 10.3389/fcvm.2026.1897722 (PMC13547249; doi:10.3389/fcvm.2026.1897722)
Supplement: Supplementary file 1 [file Table1.docx]

Supplementary Table S1. Detailed extraction of included studies with exposure contrast, effect estimates, and adjustment covariates

| Study | Outcome | TMAO Exposure Contrast | Effect Estimate (95% CI) | Adjustment Covariates | Analysis |
| --- | --- | --- | --- | --- | --- |
| Suzuki et al., 2016 | Incident AF | High vs. low TMAO (median 5.6 µM) | OR 1.38 (1.07–1.76) | Age, sex, BMI, diabetes, hypertension, smoking, eGFR, NT‑proBNP | OR |
| Zuo et al., 2018 | Incident AF | Per 1‑SD increase in log‑transformed plasma choline (TMAO precursor) | OR 1.13 (1.08–1.19) | Sex, age, BMI, diabetes, hypertension, smoking; HUSK additionally: eGFR, fasting status, education, serum folate, CRP | OR |
| Svingen et al., 2018 (WECAC) | Incident AF | Highest vs. lowest TMAO category (cut‑offs not specified) | OR 1.49 (1.19–1.86) | Model 3: age, sex, hypertension, diabetes, smoking, BMI, eGFR, CRP, HF history, LVEF, LVEDP, CAD extent, alcohol intake, valvular disease/MI/revascularization history | OR |
| Svingen et al., 2018 (HUSK) | Incident AF | Per 1‑SD increase in log‑transformed TMAO | HR 1.14 (1.03–1.26) | Model 2: age, sex, hypertension, diabetes, smoking, BMI | HR |
| Stubbs et al., 2019 | Incident AF | Q5 (>155.2 µM) vs. Q1 (≤56.6 µM) | OR 1.81 (0.94–2.76) | Age, sex, BMI, systolic BP, albumin, race, dialysis vintage, smoking, CVD history, PCI, stroke, MI, BUN, trial design factors | OR |
| Zhou et al., 2020 | Incident AF | Q4 (>7.92 µmol/L) vs. Q1 (<2.83 µmol/L) | OR 1.64 (1.22–2.20) | Model 3: age, sex, BMI, hypertension, diabetes, hyperlipidemia, NYHA class, LVEF, log NT‑proBNP, eGFR, log hsCRP | OR |
| Papandreou et al., 2021 | Incident AF | Q4 vs. Q1 TMAO quartile | OR 1.02 (0.72–1.44) | MV2: smoking, family CHD history, physical activity, alcohol, BMI, intervention group, hypertension, dyslipidemia, type 2 diabetes, lipid‑lowering/antihypertensive/antidiabetic drugs | OR |
| Kinugasa et al., 2021 | Incident AF | High (>20.37 µmol/L) vs. low (≤20.37 µmol/L) TMAO | OR 0.95 (0.48–1.81) | Propensity score: age, sex, HF hospitalization history, hemoglobin, BUN, eGFR, free carnitine, acylcarnitine, acylcarnitine/free carnitine ratio | OR |
| Nenna et al., 2024 | Postoperative AF | TMAO ≥61.8 ng/mL vs. <61.8 ng/mL | OR 2.88 (1.35–6.15) | Preoperative hemoglobin, TAPSE | OR |
| Meng et al., 2024 | AF recurrence after catheter ablation | Per 1 µmol/L TMAO increase | HR 1.78 (1.43–2.21) | Age, persistent AF, left atrial diameter | HR |

Abbreviation: AF, atrial fibrillation; BMI, body mass index; BP, blood pressure; BUN, blood urea nitrogen; CAD, coronary artery disease; CRP, C‑reactive protein; CVD, cardiovascular disease; eGFR, estimated glomerular filtration rate; HF, heart failure; hsCRP, high‑sensitivity CRP; LVEDP, left ventricular end‑diastolic pressure; LVEF, left ventricular ejection fraction; MI, myocardial infarction; NT‑proBNP, N‑terminal pro‑B‑type natriuretic peptide; NYHA, New York Heart Association; PCI, percutaneous coronary intervention; TAPSE, tricuspid annular plane systolic excursion.
